# Prediction of disease progression in individuals with subjective cognitive decline using brain network analysis

**DOI:** 10.1111/cns.14859

**Published:** 2024-07-15

**Authors:** Simin Deng, Si Tan, Xiaojing Song, Xinyun Lin, Kaize Yang, Xiuhong Li, for the Alzheimer's Disease Neuroimaging Initiative

**Affiliations:** ^1^ School of Public Health (Shenzhen) Shenzhen Campus of Sun Yat‐sen University Shenzhen Guangdong China; ^2^ Department of Rehabilitation Medicine Dongguan Eighth People's Hospital Dongguan Guangdong China; ^3^ School of Public Health (Guangzhou) Sun Yat‐sen University Guangzhou Guangdong China

**Keywords:** brain functional networks, early identification, graph theory, SCD

## Abstract

**Objective:**

The objective of this study is to explore potential differences in brain functional networks at baseline between individuals with progressive subjective cognitive decline (P‐SCD) and stable subjective cognitive decline (S‐SCD), as well as to identify potential indicators that can effectively distinguish between P‐SCD and S‐SCD.

**Methods:**

Alzheimer's Disease Neuroimaging Initiative (ADNI) database was utilized to enroll SCD individuals with a follow‐up period of over 3 years. This study included 39 individuals with S‐SCD, 15 individuals with P‐SCD, and 45 cognitively normal (CN) individuals. Brain functional networks were constructed based on the AAL template, and graph theory analysis was performed to determine the topological properties.

**Results:**

For global metric, the S‐SCD group exhibited stronger small‐worldness with reduced connectivity among nearby nodes and accelerated compensatory information transfer capacity. For nodal efficiency, the S‐SCD group showed increased connectivity in bilateral posterior cingulate gyri (PCG). However, for nodal local efficiency, the P‐SCD group exhibited significantly reduced connectivity in the right cerebellar Crus I compared with the S‐SCD group.

**Conclusion:**

There are differences in brain functional networks at baseline between P‐SCD and S‐SCD groups. Furthermore, the right cerebellar Crus I region may be a potentially useful brain area to distinguish between P‐SCD and S‐SCD.

## Graphical abstract

Exploring baseline brain functional network differences and identifying indicators for effectively distinguishing progressive subjective cognitive decline (P‐SCD) and stable subjective cognitive decline (S‐SCD).

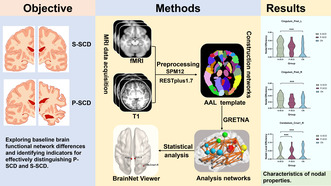

## 1 INTRODUCTION

Alzheimer's disease (AD) is the most common cause of dementia and is associated with substantial healthcare burden.^1^ Its prevalence is consistently increasing, with recent studies from the United States demonstrating that one in nine individuals aged ≥65 years have AD.^2^ AD has a profound impact on the quality of life of patients, often leading to lifelong disability. Notably, it is the fifth leading cause of death among individuals aged ≥65 years, underscoring its significance as a global public health and clinical issue.

Early identification and treatment are the most effective strategies for improving the prognosis of neurodegenerative diseases. Recent studies have found that AD may be present for 20 years or longer before symptoms appear; this time period can serve as a valuable window for intervention.^3^ Decline in cognitive abilities or worsening of memory is one of the earliest warning signs of AD, which can help identify individuals at high risk of developing AD, other dementias, or mild cognitive impairment (MCI).^4^ Subjective cognitive decline (SCD) is one of the earliest symptoms that prompt individuals to seek medical help. It refers to subjective changes in cognitive abilities experienced by individuals, unrelated to cognitive tests, physician diagnosis, or observations by others.^5^ However, not all individuals experiencing SCD develop dementia. Previous studies^6^, ^7^ have shown that only a small proportion of individuals in the SCD group and healthy control group develop objective cognitive decline (OCD), including AD dementia or MCI. Furthermore, SCD individuals may experience symptoms for approximately 15 years before the onset of MCI. In turn, MCI may occur several years before the onset of AD.^8^ Therefore, early identification of individuals with SCD who may progress, namely progressive SCD (P‐SCD), is particularly important.

Due to the absence of OCD in SCD, it is not possible to identify early progression of SCD based on cognitive assessment scales. To further explore the mechanisms underlying disease onset and progression, several neuroimaging techniques, such as resting‐state functional magnetic resonance imaging (rs‐fMRI),^9^, ^10^ have been used to assess the brain structure and functional alterations in SCD individuals. In particular, rs‐fMRI is an effective approach for the prediction of disease progression, and measurements of brain functional networks may be regarded as potential intermediate biomarkers for SCD.^11^ Previous studies by Xu et al. and Wang et al.^12^, ^13^ have found differences in functional connectivity of the default mode network (DMN) between SCD individuals and healthy controls. Additionally, Dillen et al.^14^ reported higher functional connectivity between the posterior cingulate gyri (PCG) and the frontal lobe in SCD individuals. Another study by Li et al.,^15^ using topological structure analysis, found that SCD individuals have lower centrality in the ventral area but higher centrality in bilateral hippocampi and the left fusiform gyrus. Notably, the aforementioned studies were cross‐sectional, thereby limiting their ability to examine the dynamic brain changes. It is not possible to determine whether SCD will further progress to P‐SCD. Longitudinal studies can evaluate the brain changes during different SCD stages, but previous longitudinal studies on SCD have mainly focused on predicting factors^16^ and modifiable protective factors^7^ for OCD progression in SCD. Viviano and Damoiseaux^17^ speculated that the trajectory of brain changes from SCD to OCD is non‐linear and proposed that enhanced network functional connectivity is a feature of early SCD, which gradually transitions to decreased functional connectivity over time. However, longitudinal studies are needed for verification. Tijms et al.^18^ found that, based on abnormal amyloid, approximately 50% of predementia AD patients will develop dementia within 3 years. Therefore, the present study categorized SCD individuals into stable SCD (S‐SCD) and progressive SCD (P‐SCD) groups based on progression to MCI or AD and followed them over a 3‐year period. The objective of our study was to explore the differences in brain functional networks at baseline between S‐SCD, P‐SCD, and cognitively normal (CN) individuals.

We proposed the following hypotheses based on the above content: There are significant differences in brain functional networks at baseline between individuals with P‐SCD and S‐SCD, and certain network properties among these differences can serve as effective markers for predicting future cognitive decline. To validate these hypotheses, we obtained data from the Alzheimer's Disease Neuroimaging Initiative (ADNI; adni.loni.usc.edu), which includes 62 SCD individuals who were followed up for more than 3 years. After 3 years, the clinical outcomes were used to classify individuals into the S‐SCD group (*n* = 41) or the P‐SCD group (*n* = 21). Furthermore, we randomly selected rs‐fMRI data from 45 CN individuals and constructed brain functional networks for all three groups. Subsequently, we analyzed the differences in topological structure and functional connectivity among these groups. This study innovatively applies graph theory to analyze brain functional networks from the ADNI database, identifying novel biomarkers that enhance the prediction and management of SCD.

## 2 METHODS

### 2.1 Study participants

This study collected raw data from the ADNI2 database, which includes comprehensive information. The process of extracting individuals from ADNI involved setting clear selection criteria, securing access through the official website, filtering eligible individuals, extracting relevant data, conducting quality control, and finally analyzing the data. However, the number of participants with SCD was relatively small because the recruitment started from ADNI2. Baseline data were available for 106 participants, whereas 62 participants had undergone follow‐up visits for longer than 3 years. This study included 62 individuals with SCD, categorizing them into S‐SCD (*n* = 41; Clinical Dementia Rating, CDR = 0) or P‐SCD (*n* = 21; CDR > 0) based on the 3‐year clinical outcomes. Additionally, 45 CN individuals were also enrolled. Eight participants were excluded due to low‐quality images. The study protocol was approved by the ethics committee of the Institutional Review Board (IRB), and all patients provided written informed consent. Before initiating the analysis, approval was secured from the local ethics committee of Dongguan Eighth People's Hospital (Dongguan Children's Hospital) under the reference AF/SC‐15/01.0.

For the SCD group, the following inclusion criteria^5^ were used: (1) Individual and caregiver confirmation of SCD, with a score ≥ 16 on the Cognitive Change Index for the first 12 items; (2) normal memory function on the Wechsler Memory Scale‐Logical Memory (WMS‐LM)^19^: (a) ≥16 years of education with score ≥ 9; (b) 8–15 years of education with score ≥ 5; (c) 7 years of education with score ≥ 3; (3) Mini‐Mental State Examination (MMSE)^20^ score of 24–30 (inclusive); (4) CDR^21^ score of 0; (5) normal cognition, including intact cognitive function and activities of daily living; (6) geriatric Depression Scale^22^ score of 6; (7) Age between 55 and 90 years (inclusive); (8) presence of a caregiver who is available to spend at least 10 h per week in communication; (9) normal visual and auditory functions as well as ability to cooperate with neuropsychological assessments; (10) good general health; (11) willingness to participate in longitudinal neuroimaging studies; (12) Hachinski Ischemic Score^23^ of 4; (13) education or work experience of at least 6 years to exclude mental retardation; (14) absence of speech disorders; (15) willingness to undergo neuroimaging examinations and absence of contraindications; (16) agreement to blood collection for biomarker testing; and (17) agreement to undergo lumbar puncture for cerebrospinal fluid collection. The CN group included individuals with no cognitive impairment or decline as reported by themselves or their caregivers, with the remaining criteria similar to those for the SCD group.

We excluded individuals with (1) neurodegenerative diseases, such as Parkinson's disease, multiple sclerosis, epilepsy, multi‐infarct dementia, hydrocephalus, Huntington's disease, supranuclear palsy, brain tumors, traumatic brain injury, subdural hematoma, and structural abnormalities of the brain; (2) infections, infarctions, or other focal lesions detected by magnetic resonance imaging (MRI); (3) implants or foreign bodies; (4) severe major depressive disorder or bipolar disorder in the past year; (5) current use of medications for obsessive‐compulsive disorder or attention‐deficit disorder; (6) history of schizophrenia; (7) history of alcohol or substance abuse or dependence in the past 2 years; (8) severe systemic diseases; (9) abnormal vitamin B12 levels; (10) current use of specific medications, including cholinergic antidepressants, warfarin, chronic anti‐anxiety or sedative‐hypnotic medications, and other exclusionary medications; or (11) participation in clinical studies involving neuropsychological measures being collected more than one time per year.

### 2.2 MRI data acquisition and preprocessing

The study participants were selected from the baseline of ADNI2 and were required to have both structural MRI and rs‐fMRI data. A 3.0‐Tesla MRI scanner was used for scanning. Structural MRI was acquired using a T1‐weighted 3D MPRAGE sequence, whereas rs‐fMRI was acquired using an echo planar imaging (EPI) sequence.

For structural MRI, the scanning parameters were as follows: repetition time of 2300 ms, echo time of 3.0 ms, flip angle of 9.0°, spatial resolution of 1.1 × 1.1 × 1.2 mm^3^, 170 slices, slice thickness of 1.2 mm, and an inversion time of 900 ms. For the rs‐fMRI, the scanning parameters were as follows: repetition time of 3000 ms, echo time of 30.0 ms, flip angle of 90.0°, spatial resolution of 3.4 × 3.4 × 3.4 mm^3^, 48 slices, and a slice thickness of 3.4 mm.

The rs‐fMRI image data were preprocessed using the RESTplus1.7 software (http://www.restfmri.net/forum/RESTplusV1.7)^24^ based on the Statistical Parametric Mapping (SPM12) software (https://www.fil.ion.ucl.ac.uk/spm/software/spm12) in the MATLAB R2013b platform (The MathWorks Inc., Natick, MA, USA). The preprocessing involved the following steps^25^: conversion of raw data from the DICOM format to the NIFTI format; removal of the first 10 unstable time points; time slice correction using interpolation to align each scan to the actual result at a certain time, using odd‐numbered slices as the starting point for interleaved scanning; head motion correction with a threshold of 3 mm for displacement and 3° for rotation, excluding participants who exceed the threshold; spatial standardization using EPI registration to align the data to the standard space; removal of linear trends to correct for linear drift potentially caused by factors such as machine heating; filtering with a valid frequency range of 0.01–0.08 Hz; and application of regression covariates including white matter, cerebrospinal fluid, and head motion noise.

### 2.3 Construction and analysis of functional networks

The AAL template was applied to the brain^26^ by dividing the brain into 90 areas and the cerebellum into 26 areas. Each area served as a node for the brain network. The GRETNA software (The graph‐theoretical network analysis toolkit, www.nitrc.org)^27^ based on MATLAB was used to evaluate the connectivity matrix between paired regions of interest using the Pearson correlation coefficient for the average time series. Then, the connectivity matrix of each participant was binarized^28^ to construct the functional network. Graph theory analysis was used to evaluate the brain functional network characteristics of study participants. A sparsity range of 0.05–0.16 with an interval of 0.01 was applied, as more than 90% of participants exhibited small‐worldness with a sparsity ≤0.16. The iteration was performed 1000 times.

### 2.4 Network topological properties

The network topology properties of the resting‐state functional network can be categorized into global network characteristics and node characteristics within the brain.^29^ Global network characteristics encompass several features, including the small‐worldness coefficient (σ), clustering coefficient (CP), shortest path length (LP), normalized clustering coefficients (γ), and normalized characteristic path length (λ). Additionally, efficiency is an important parameter that is further categorized into global efficiency (Eg) and local efficiency (Eloc). Finally, assortativity is also considered at the global network level. On the other hand, node characteristics focus on individual nodes within the network and include node CP, node LP, node Eg, node Eloc, degree centrality (DC), and betweenness centrality (BC).

### 2.5 Statistical analysis

The general characteristics of the P‐SCD, S‐SCD, and CN groups were analyzed using SPSS 26.0 software. The Shapiro–Wilk test was used to assess the normality of the dataset. In instances where the dataset deviated from normal distribution, the Kruskal–Wallis rank sum test was utilized as a non‐parametric alternative. One‐way analysis of variance (ANOVA) was used to assess differences among groups in terms of education, age, cerebrospinal fluid biomarkers, and neuropsychological evaluation results. To account for multiple comparisons, Bonferroni correction was applied to the significant differences identified. Additionally, gender differences were evaluated using the χ^2^ test, and Dunn multiple comparison correction was applied to significant differences. Furthermore, network properties were analyzed using one‐way ANOVA *F*‐test and two‐sample *t*‐test in the Global and Nodal Metric Comparison module of GRETNA. For global metric, one‐way ANOVA was conducted for the three groups of data, followed by post‐hoc analysis using independent samples *t*‐test. Detailed analysis was facilitated using SPSS, beginning with the assessment of variance homogeneity among the groups. In cases of unequal variances, Welch's ANOVA was used, succeeded by post‐hoc comparisons to pinpoint specific differences between groups. For situations where variances were equal, the Least Significant Difference (LSD) test was applied. Conversely, the Games‐Howell test was utilized to address scenarios of variance inequality. For nodal metric, one‐way ANOVA was conducted for the three groups of data, followed by independent samples *t*‐test, which revealed significant results, and multiple comparison correction (FWE correction). To delve deeper into the relationship between cognitive assessments and network topological properties, we began our analysis with Pearson correlation analysis. Tracts that exhibited significant correlations were then incorporated into a stepwise multiple linear regression model, where cognitive scores served as the dependent variable, and network topological properties were the independent variables, adjusted for age, gender, and years of education. *p*‐Value < 0.05 was considered statistically significant.

## 3 RESULTS

### 3.1 Demographic characteristics

As a result, 45, 39, and 15 participants were included in the CN, S‐SCD, and P‐SCD groups, respectively. Table 1 summarizes the baseline characteristics of the study participants. There were no significant differences between the groups in terms of age, gender, or educational status (all *p* > 0.05). The P‐SCD group demonstrated significantly higher 13‐item AD assessment scale scores compared with the S‐SCD and CN groups (*p* = 0.033). Conversely, the WMS‐LM scores were significantly lower in the P‐SCD group compared with the S‐SCD and CN groups (*p* = 0.015). Therefore, these two indicators were incorporated as covariates in subsequent image processing. The significance threshold for these covariates was set at *p* < 0.05.

**TABLE 1:** Differences in demographic characteristics, neuropsychological assessment, and cerebrospinal fluid biomarkers among the three groups ($\bar{x}$ ± SD).

Demographic	CN (*n* = 45)	S‐SCD (*n* = 39)	P‐SCD (*n* = 15)	*F*/χ^2^	*p*	Post‐hoc analysis
Age (years)	73.14 ± 6.07	70.75 ± 4.97	72.11 ± 5.84	1.962	0.146	–
Females/Males	21/24	23/16	8/7	1.707	0.191	–
Education	16.38 ± 2.64	16.36 ± 2.56	16.20 ± 2.68	0.027	0.973	–
ADAS13 score	9.06 ± 4.05	8.51 ± 4.32	12.13 ± 6.73	3.533	**0.033**	S‐SCD < CN S‐SCD < P‐SCD
RAVLT score	44.47 ± 10.21	46.00 ± 8.04	39.40 ± 7.60	2.897	0.060	–
MMSE score	29.00 ± 0.00	29.00 ± 0.97	28.80 ± 1.57	0.347	0.708	–
WMS‐LM score	13.04 ± 2.55	13.59 ± 3.16	11.00 ± 3.07	4.419	**0.015**	S‐SCD > CN S‐SCD > P‐SCD
TMT‐B score	78.80 ± 41.58	77.51 ± 36.18	108.80 ± 76.02	2.775	0.067	–
Aβ1‐42	1142.31 ± 433.75	1312.77 ± 399.86	1194.08 ± 472.10	1.599	0.208	–
t‐tau	210.87 ± 66.85	231.60 ± 91.48	215.53 ± 85.12	0.687	0.506	–
p‐taul81	20.73 ± 11.03	20.93 ± 9.47	19.19 ± 9.17	0.105	0.900	–

*Note*: Units for cerebrospinal fluid biomarkers are ng/L. Data are presented as mean ± standard deviation. *F*‐values were obtained from analysis of variance test; χ^2^ test was used.

Abbreviations: ADAS13, Alzheimer's disease assessment scale; MMSE, mini‐mental state examination; RAVLT, Rey Auditory Verbal Learning Test; TMT‐B, Trail Making Test Part B; WMS‐LM, Wechsler Memory Scale‐Logical Memory.

The bold values indicate statistically significant differences between groups, with *p* < 0.05.

### 3.2 Global metric

**FIGURE 1:**
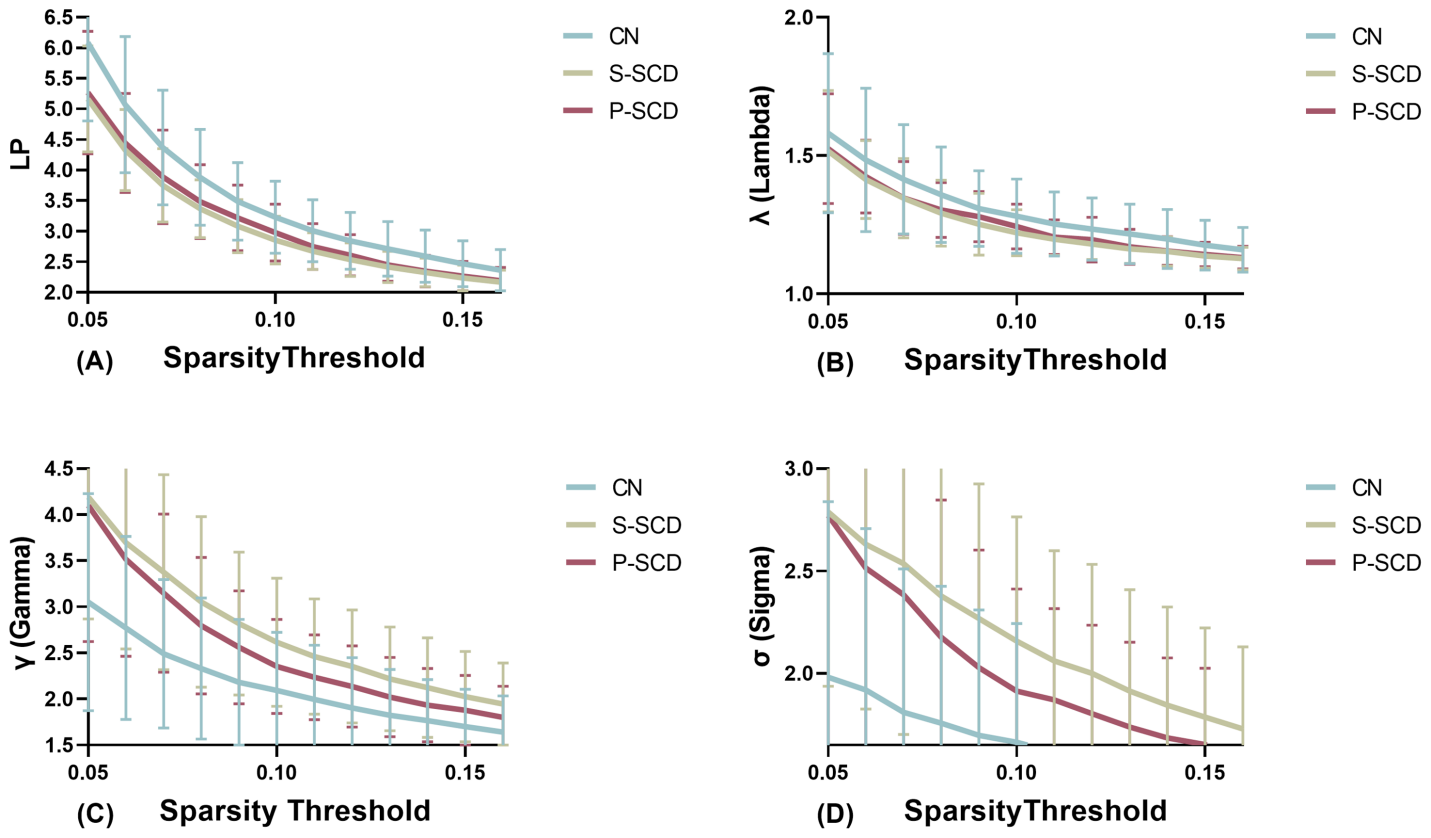
Small‐worldness of the whole‐brain resting‐state functional network among the three groups at a sparsity rating of 0.05–0.16. (A) LP indicates the shortest path length. The AUCs of LP were significantly lower in the S‐SCD group compared with the CN group. The P‐SCD group exhibited AUC values intermediate between the S‐SCD and CN groups, but there were no statistically significant differences among the groups. (B) λ indicates the normalized characteristic path length. The AUC values of λ were significantly lower in the S‐SCD group compared with the CN group. The P‐SCD group exhibited values intermediate between the S‐SCD and CN groups, but there was no statistically significant difference between the groups. (C) γ indicates the normalized clustering coefficient. The AUC values of γ were significantly higher in the S‐SCD group compared with the CN group. Similarly, the P‐SCD group had values intermediate to those of the S‐SCD and CN groups, with no statistically significant differences from the other groups. (D) σ indicates the small‐worldness coefficient. The AUC values of σ were significantly higher in the S‐SCD group compared with the CN group. Similarly, the AUC values of the P‐SCD group were intermediate between the S‐SCD and CN groups, without statistically significant differences between the groups. CN, cognitively normal; LP, shortest path length; P‐SCD, progressive subjective cognitive decline; S‐SCD, stable subjective cognitive decline; γ, normalized clustering coefficient; λ, normalized characteristic path length; σ, small‐worldness coefficient.

**FIGURE 2:**
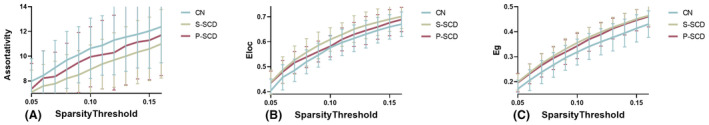
Whole‐brain resting‐state functional network of the three groups at a sparsity rating of 0.05–0.16. (A) The AUC value of assortativity was lower in the S‐SCD group compared with the P‐SCD and CN groups. (B) Eloc indicates the local efficiency. The AUC values of Eloc were higher in the S‐SCD group compared with the CN group. The P‐SCD group showed AUC values intermediate between the S‐SCD and CN groups, but without statistically significant differences among the groups. (C) Eg indicates the global efficiency. The AUC values of Eg were higher in the S‐SCD group compared with the CN group. The P‐SCD group showed AUC values intermediate between the S‐SCD and CN groups, but without statistically significant differences among the groups. CN, cognitively normal; Eg, global efficiency; Eloc, local efficiency; P‐SCD, progressive subjective cognitive decline; S‐SCD, stable subjective cognitive decline.

Using the area under the curve (AUC) as an independent variable, we conducted inter‐group comparisons with a sparsity range of 0.05–0.16 and an interval of 0.01. Table 2 presents a comparison of whole‐brain network characteristics among the CN, S‐SCD, and P‐SCD groups. Within the wider sparsity range of 0.05–0.16, the AUC values of LP (*p* = 0.002) and λ (*p* = 0.004, Figure 1) were significantly lower in the S‐SCD group compared with the CN group. The P‐SCD group exhibited intermediate values between the S‐SCD and CN groups, albeit without statistically significant differences between the groups. Moreover, the AUC values of γ (*p* = 0.002) and σ (*p* = 0.001, Figure 1) were significantly higher in the S‐SCD group compared with the CN group. Similarly, the AUC values for the P‐SCD group were intermediate between that of the S‐SCD and CN groups, with no statistically significant differences between the groups. Additionally, the AUC values of Eg (*p* = 0.002) and Eloc (*p* = 0.008, Figure 2) were higher in the S‐SCD group compared with the CN group. The P‐SCD group demonstrated values intermediate between the S‐SCD and CN groups, with no statistically significant differences between the groups. Furthermore, the AUC of the z‐score value of assortativity was lower in the S‐SCD group compared with the P‐SCD and CN groups (Figure 2, *p* = 0.025). Additionally, the AUC of the z‐score value for P‐SCD group was intermediate between the S‐SCD and CN groups, without statistically significant differences between the groups. Finally, the inclusion of regression covariates did not significantly alter the results.

**TABLE 2:** Characteristics of global properties among the three groups ($\bar{x}$ ± SD).

Global properties	CN (*n* = 45)	S‐SCD (*n* = 39)	P‐SCD (*n* = 15)	*F*	*p*	Post‐hoc analysis
CP	0.0506 ± 0.0036	0.0524 ± 0.0042	0.0508 ± 0.0032	2.346	0.102	–
LP	0.3787 ± 0.0664	0.3323 ± 0.0408	0.3415 ± 0.0511	6.993	**0.002**	S‐SCD < CN
*λ*	0.2341 ± 0.0701	0.2981 ± 0.0816	0.2753 ± 0.6577	5.816	**0.004**	S‐SCD > CN
*γ*	0.1430 ± 0.1418	0.1368 ± 0.0853	0.1380 ± 0.0799	3.817	**0.002**	S‐SCD < CN
*σ*	0.1821 ± 0.0618	0.2384 ± 0.0655	0.2195 ± 0.060	7.489	**0.001**	S‐SCD < CN
Eg	0.0351 ± 0.0053	0.0391 ± 0.004	0.0383 ± 0.0048	6.769	**0.002**	S‐SCD > CN
Eloc	0.0627 ± 0.0050	0.0665 ± 0.0053	0.0646 ± 0.0047	5.074	**0.008**	S‐SCD > CN
Hierarchy	−0.2217 ± 0.1033	−0.1862 ± 0.0837	−0.1680 ± 0.0578	2.271	0.110	–
Assortativity (z‐score)	1.1567 ± 0.2545	1.0002 ± 0.2168	1.0816 ± 0.2727	3.865	**0.025**	S‐SCD > CN
Synchronization	−0.0614 ± 0.0597	−0.0955 ± 0.0981	−0.1324 ± 0.1446	3.090	0.051	–

*Note*: Data presented as mean ± standard deviation.

Abbreviations: CP, clustering coefficient; Eg, global efficiency; Eloc, local efficiency; LP, shortest path length; *γ*, standardization clustering coefficient; λ, standardization of path length; σ, small‐worldness coefficient.

The bold values indicate statistically significant differences between groups, with *p* < 0.05.

### 3.3 Nodal metric

**FIGURE 3:**
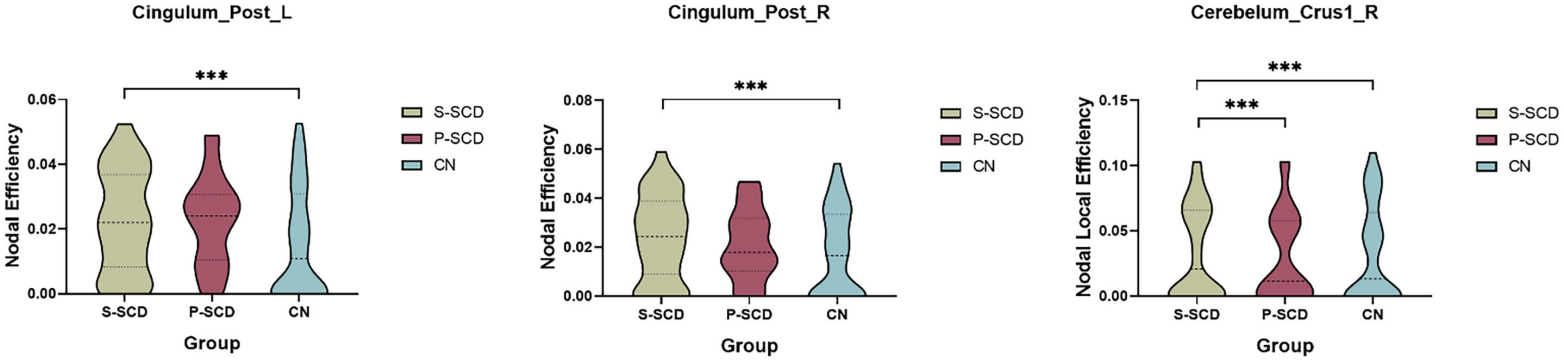
Differences in nodal local efficiency among the three groups. Compared with the CN group, both the S‐SCD and P‐SCD groups exhibited changes in the Eloc of nodes in bilateral PCG. In particular, the S‐SCD group demonstrated a significant increase in the Eloc in this area. The P‐SCD group exhibited significantly lower connectivity in the right cerebellum Crus I compared with the S‐SCD group. CN, cognitively normal; Eloc, local efficiency; PCG, posterior cingulate gyrus; P‐SCD, progressive subjective cognitive decline; S‐SCD, stable subjective cognitive decline. *** indicates *p* < 0.05, indicating statistically significant differences between groups.

**FIGURE 4:**
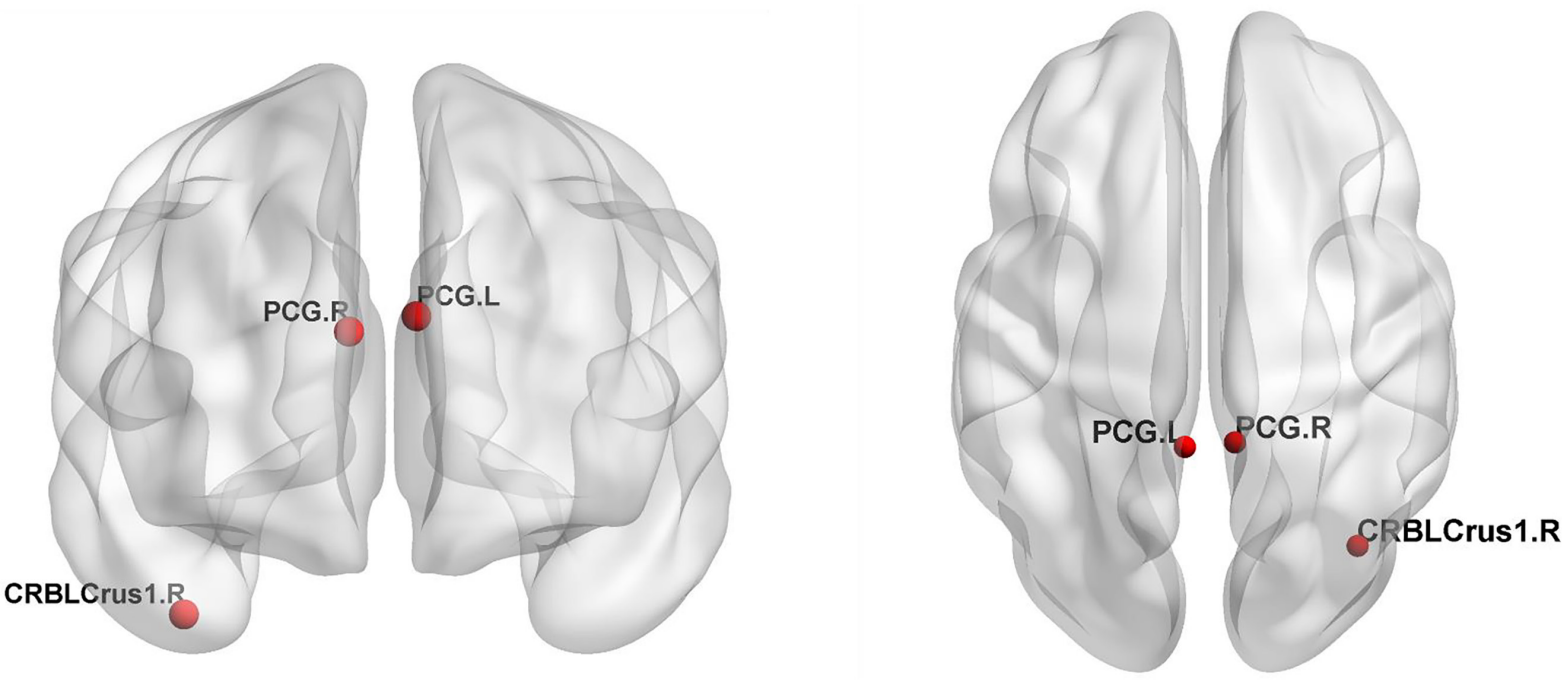
Characteristics of nodal properties. CRBLCrus 1.R, right cerebellar Crus I; PCG.L, posterior cingulate gyrus, left; PCG.R, posterior cingulate gyrus, right.

Compared with the CN group, both the S‐SCD and P‐SCD groups exhibited changes in the Eloc of nodes in bilateral PCG. In particular, the S‐SCD group demonstrated a significant increase in the Eloc in this region (Figures 3 and 4, *p* = 0.000). The P‐SCD group also showed a similar increasing trend, although it did not reach statistical significance (*p* = 0.000). A significant difference was observed in the connectivity between the two SCD groups. The P‐SCD group exhibited significantly lower connectivity in the right cerebellum Crus I compared with the S‐SCD group (Figures 3 and 4, *p* = 0.000). No significant differences were observed between the SCD groups when considering regression covariates.

### 3.4 Association between cognitive assessments and network topological properties

Table 3 presents the results of a linear regression analysis investigating the associations between cognitive scores and network topological characteristics. This analysis revealed that the topological properties of λ were significantly positively correlated with the RAVLT scores (β = 1.143, *p* = 0.023). Similarly, the topological properties of Eloc were significantly negatively correlated with the TMT‐B scores (β = −0.539, *p* = 0.043).

**TABLE 3:** Multiple linear regression analysis of various cognitive assessments and network topological properties.

Dependent variable	Variables included in the model standardized	Coefficients β	*t*‐Value	*p*‐Value
RAVLT score	*λ*	1.143	2.325	**0.023** *
TMT‐B score	Eloc	−0.539	−2.061	**0.043** *

*Note*: Initial analyses identified correlations between cognitive scores and network topological properties. Variables exhibiting significant correlations (*p* < 0.05) were subjected to further evaluation through stepwise multiple linear regressions, adjusted for age, gender, and educational background. *p*‐values in bold indicate tracts significantly correlated with cognitive functions.

Abbreviations: Eloc, local efficiency; RAVLT, Rey Auditory Verbal Learning Test; TMT‐B, Trail Making Test Part B; λ, standardization of path length.

* *p* < 0.05, indicating statistically significant differences between groups.

## 4 DISCUSSION

This study investigated whether there are differences in brain functional networks between P‐SCD and S‐SCD at baseline, and whether certain network properties among these differences can serve as effective markers for predicting future cognitive decline. Our findings indicated that, the S‐SCD groups exhibited stronger small‐worldness compared with the CN group. Furthermore, the S‐SCD groups demonstrated increased Eloc of bilateral PCG connections. However, the P‐SCD group exhibited significantly reduced connectivity in the right cerebellum Crus I compared with the S‐SCD group. These results align with previous findings, such as those by Viviano and Damoiseaux,^17^ which confirmed brain functional network differences between P‐SCD and S‐SCD at baseline. Additionally, we demonstrated that the right Crus I of the cerebellum is a potentially useful region for distinguishing between the two. By analyzing network topological properties, this research provides novel insights and significant clinical implications for the early detection and differentiation of SCD.

With regard to small‐worldness, the statistically significant indicators are LP, λ, γ, and σ. LP is the average shortest path length from node *i* to all other nodes within the network, which represents the closeness of network connections. The LP value of the S‐SCD groups was lower than that of the CN group, indicating faster information transfer between network nodes. λ is the normalized LP, which is the ratio of the network's actual LP value to the average LP value of 500 random networks. The value of the S‐SCD groups was lower than that of the CN group. σ is the small‐world coefficient, defined as the ratio of standardized CP to standardized LP. Small‐worldness is indicated by σ = γ/λ > 1, with larger values correlating with greater small‐worldness. The σ value of the S‐SCD groups was higher than that of the CN group. Statistically significant differences were observed in both Eg and Eloc. The Eg value in the S‐SCD groups was higher than that in the CN group. Eg is an indicator of the efficiency of information propagation between nodes in the brain network. The Eloc value in the S‐SCD groups was higher than that in the CN group. Eloc is a measure of the local efficiency of the entire brain network, representing the global efficiency of each subnetwork (Gi) and the information transmission capacity of the nodes in the network. There was a statistically significant difference between the groups in terms of assortativity, with the assortativity value being lower in the S‐SCD groups than the CN group. A lower assortativity value indicates a decrease in the mutual connectivity among similar nodes. Additionally, the observed differences between the groups remained significant regardless of whether the covariates were included, indicating that these differences are robust to the inclusion of covariates. With regards to global metric, the S‐SCD group exhibited stronger small‐worldness with reduced connectivity among nearby nodes and accelerated compensatory information transfer capacity.

Based on the aforementioned findings related to brain topology, we found that, compared with the CN group, the S‐SCD group showed greater small‐worldness and a lower mutual connectivity among similar nodes, indicating an increase in compensatory information transmission capability, which is consistent with our hypothesis of altered compensatory brain topology in SCD. These findings align with those of Chen et al.,^11^ who also observed increased small‐worldness in SCD. However, the P‐SCD group showed certain changes that were not statistically significant, likely due to the small sample size. Our results differ from those of Xu et al.,^12^ mainly due to differences in the inclusion criteria and research methods. Pereira et al.^30^, ^31^, ^32^ found altered small‐worldness in AD patients, characterized by a more random gray matter network and lower characteristic path length. Verfaillie et al.^18^, ^33^ found similar changes in gray matter network patterns in SCD individuals as in AD patients, with SCD individuals exhibiting a more random gray matter network compared with the normal control group, and the disruption of network characteristics being associated with a sharp decline in overall cognitive ability and a high risk of disease progression. These studies highlight the variability in small‐worldness and network characteristics across different stages of cognitive decline. However, Kate et al.^34^ found higher overall amyloid deposition and lower clustering of gray matter structural networks in SCD individuals, with fewer small‐world characteristics. This discrepancy may be due to methodological differences and highlights the complexity of brain network changes in SCD. Our study specifically found that the S‐SCD group exhibited greater small‐worldness, likely due to compensatory mechanisms, which is consistent with our hypothesis and supports the notion of enhanced network efficiency in early stages of cognitive decline.

Compared with the CN group, the S‐SCD group showed higher efficiency in bilateral posterior cingulate nodes in terms of node characteristics of functional network connections within the brain. PCG is a highly connected and metabolically active brain region. It is a key node in the DMN and plays a crucial role in self‐referential and prospective cognitive processes, as well as in unconstrained “rest” periods when the brain activity increases. However, it exhibits high heterogeneity and may directly regulate the attentional focus.^35^ This aligns with findings from previous studies, which highlight the PCG's importance in cognitive processes and its vulnerability in neurodegenerative diseases.^36^ Reduced metabolism in the PCG is an early feature of AD and typically occurs before a definite clinical diagnosis is made.^37^ In AD patients, metabolic abnormalities in the PCG are associated with amyloid deposition and brain atrophy. Furthermore, these patients demonstrate reduced functional connectivity within the DMN, particularly affecting connectivity between the PCG and hippocampus.^38^ These findings are consistent with our observation of increased efficiency in the PCG in the S‐SCD group, suggesting compensatory mechanisms may be at play. The genetic status of apolipoprotein E (*APOE*) has a significant impact on the functional connectivity pattern of the PCG. Moreover, increased functional connectivity within the DMN has been observed in healthy young individuals carrying the *APOE*ε4 allele, which may be related to increased neural activity required to compensate for network inefficiencies.^39^ Amyloid deposition in the PCG is more common in AD patients, which may be due to the neural cascade reaction caused by the high metabolic activity in the central network of the brain.^40^, ^41^ These aspects further support the relevance of our findings in the S‐SCD group, indicating that increased PCG efficiency could serve as a biomarker for early detection and differentiation of cognitive decline.

Among studies of functional connectivity in MCI patients and SCD individuals, Kang et al.^42^ found greater functional connectivity in PCG in patients with early‐stage MCI compared with the CN group and patients with late‐stage MCI. However, Lee et al.^43^ found that functional connectivity in the DMN was reduced in early‐stage MCI patients compared with the CN group, which was further reduced in late‐stage MCI patients. These findings suggest variability in DMN connectivity changes across different stages of cognitive decline. Zhan et al.^44^ identified a gradual reduction in inter‐network functional connectivity involving the DMN as individuals progressed from CN to late‐stage MCI. This aligns with our observation of altered connectivity patterns in the S‐SCD group. Dong et al.^45^ found that the functional connectivity in PCG was higher in SCD individuals compared with the CN group, which is consistent with our findings. The increased connectivity in the PCG may reflect its compensatory role in early SCD stages, dynamically balancing brain load or reshaping damaged brain topology through neural plasticity. Our findings suggest the PCG plays a crucial role in maintaining cognitive function during early cognitive decline, highlighting its potential as a biomarker for early detection and differentiation of SCD.

The Eloc value in the right cerebellar Crus I area is reduced in P‐SCD individuals. The right cerebellar Crus I is located in the posterior part of the cerebellum and is associated with the lateral prefrontal area, which is activated by increased cognitive load. This region, unrelated to the anterior part of the cerebellum, plays a crucial role in cognitive processes.^46^ Although the cerebellum is primarily involved in motor processing,^47^ but it also participates in cognitive processes.^48^ Functional MRI studies in healthy individuals have confirmed that motor tasks, such as finger tapping, activate lobules IV–VI in the anterior part of the cerebellum, whereas memory tasks involving attentional shifts or verbal work activate the posterior hemisphere of the cerebellum, mainly in lobules VIIA (comprised of Crus I and II) and VIIB. The cerebellar vermis is situated in lobule VII of the posterior cerebellar hemisphere.^49^ Furthermore, in AD patients, there are abundant Aβ deposits and increased numbers of microglia in the cerebellar vermis.^50^, ^51^ Bai et al.^52^ found that patients with amnestic MCI exhibit increased functional connectivity in the posterior part of the cerebellum with the temporal and parietal cortices at baseline, but this connectivity decreased after a 20‐month follow‐up. The present study demonstrated a decrease in the right Crus I node of the cerebellum in the P‐SCD group in terms of the Eloc node. This decrease may indicate early disruptions in connectivity that could signal the progression of SCD. Our study elucidates the existence of separate areas in the human cerebellum activated during cognitive and motor processes, each with different fiber tracts and connectivity patterns with the cerebral cortex. The cerebellar Crus I/II area, activated by increased cognitive load, facilitates communication with brain areas similarly activated by cognitive demands. This region's involvement in optimizing response speed suggests that reduced connectivity in the right Crus I of the cerebellum at baseline may serve as a potential indicator of SCD progression.

There are certain limitations in this study. First, we did not analyze the AD pathology, including Aβ and tau deposits, which can impact the cognitive decline and brain changes during AD progression.^53^ Second, there were significant differences in the demographic characteristics, genetic factors, and recruitment sources among the study participants. Third, there were significant differences in the follow‐up duration, with longer follow‐up periods leading to a higher risk of cognitive decline. Additionally, our analysis was limited by using single‐time point data. We recognize that multi‐time point data collection for survival analysis would yield deeper insights into disease progression. Future studies will adopt this approach to better understand cognitive decline dynamics. Finally, a limited sample size was included. Further studies with a large sample size are needed to verify our results. Given the complex interplay between neuroinflammation, neurodegeneration, genetic susceptibility, personal lifestyle choices, and psychological stress, it is crucial to explore the various factors influencing the progression of SCD in the future.

## 5 CONCLUSION

This study investigated the differences in brain functional networks between S‐SCD, P‐SCD, and CN at baseline and explored the potential indicators that can effectively distinguish between S‐SCD and P‐SCD. Compared with the CN group, the S‐SCD group exhibited greater small‐worldness and increased connectivity in bilateral PCG. However, the P‐SCD group exhibited significantly reduced connectivity in the right cerebellar Crus I compared with the S‐SCD group. The right cerebellar Crus I at baseline may be a potential predictor for SCD progression. Our study underscores the importance of analyzing network topology properties for the early prediction of SCD, providing key insights and practical clinical strategies.

## CONFLICT OF INTEREST STATEMENT

The author reports no conflicts of interest in this work.

## Data Availability

The data that support the findings of this study are available in The Alzheimer's Disease Neuroimaging Initiative, ADNI at https://adni.loni.usc.edu/. These data were derived from the following resources available in the public domain: ADNI2 database, https://adni.loni.usc.edu/.
